# On the half-life of thiocyanate in the plasma of the marine fish *Amphiprion ocellaris*: implications for cyanide detection

**DOI:** 10.7717/peerj.6644

**Published:** 2019-04-02

**Authors:** Nancy E. Breen, J. Alexander Bonanno, Sara Hunt, Julia Grossman, Jordan Brown, Hannah Nolte, Andrew L. Rhyne

**Affiliations:** 1 Department of Chemistry, Roger Williams University, Bristol, RI, USA; 2 School for the Environment, University of Massachusetts at Boston, Boston, MA, USA; 3 Department of Biology, Marine Biology, and Environmental Science, Roger Williams University, Bristol, RI, USA

**Keywords:** Cyanide fishing, Aquarium trade, HPLC, Toxicology, Thiocyanate

## Abstract

The illegal practice of using cyanide (CN) as a stunning agent to collect fish for both the marine aquarium and live fish food trades has been used throughout the Indo-Pacific for over 50 years. CN fishing is destructive to all life forms within the coral reef ecosystems where it is used and is certainly one of many anthropogenic activities that have led to 95% of the reefs in the Indo-Pacific being labeled at risk for degradation and loss. A field-deployable test for detecting fish caught using CN would assist in combating the use of this destructive practice, however, no reliable and robust test exists. Further, there is little toxicokinetic data available on marine fish to support the development of such a test, yet such data is critical to establishing the concentration range and time scale over which such a test would be viable. This study presents the first direct measurement of the half-life of the metabolite thiocyanate (SCN) after pulsed exposure to CN in a marine fish. SCN was measured in the plasma of *Amphiprion ocellaris* after exposure to 50 ppm CN for three exposure times (20, 45, and 60 s) using HPLC-UV and a C30 column pre-treated with polyethylene glycol. Plasma SCN levels observed are dose-dependent, reflecting a longer time for conversion of CN to SCN as the dose of CN increases. SCN plasma levels reached a maximum concentration (1.2–2.3 ppm) 12–20 h after exposure to CN. The half-life for the elimination of SCN was 1.01 ± 0.26 days for 45 s exposure and 0.44 ± 0.15 days for 20 s exposure. Fish were also directly exposed to SCN (100 ppm for 11 days) and the observed half-life for SCN elimination was 0.35 ± 0.07 days. Plasma SCN levels did not return to control levels, even after 41 days when exposed to CN but did return to control levels after 48 days when exposed to SCN. The similar half-lives observed for CN and SCN exposure suggests that SCN exposure can be used as a proxy for measuring the rate of SCN elimination following CN exposure. In order for plasma SCN to be used as a marker for CN exposure, these results must be extended to other species and endogenous levels of SCN in wild caught fish must be established.

## Introduction

Despite being illegal in most countries, cyanide (CN) fishing continues to be used throughout the Indo-Pacific region to capture reef fish for the marine aquarium trade (MAT) and live fish food trade (LFFT) industries (Calado et al., 2014; Davis, Murray & Katsiadaki, 2017; Losada & Bersuder, 2017). This fishing method was first used in 1957 in the US as a convenient, efficient, and economical method for preparing aquaculture ponds by removing unwanted fish (Lewis & Tarrant, 1960). Shortly thereafter, the practice was observed in the Philippines for the collection of fish harvested for the MAT with subsequent adoption by the LFFT (Rubec, 1986, 1987). As both of these industries grew, the use of CN as a means to stun and capture reef fish became widespread.

Cyanide (as HCN or the anion CN^−^, CN) fishing occurs when divers squirt aqueous solutions of CN onto coral reefs to temporarily stun reef fish making for easy capture. While thought to be more efficient than traditional capture methods, the ecological consequences of this practice are numerous (Halim, 2002). CN itself is non-selective, poisoning or killing both targeted and non-targeted fish alike. Fish that survive the exposure and are captured have a much higher mortality rate post-capture resulting in the need to harvest more fish (Rubec & Cruz, 2005). CN fishing poses a risk to the fishermen themselves who are likely to suffer adverse health effects from chronic low-level exposure during the capture process (Frey, 2013). The coral reef ecosystem and the many life forms that rely on the reef are also prone to damage or death from the exposure to CN. Finally, structural damage to the reef by the fishermen as they attempt to capture fish also occurs (Berkes & Frey, 2017). It has been estimated that over the past 50 years more than one million kilograms of CN has been used on reefs in the Philippines alone (Mak, Yanase & Renneberg, 2005). This constant and prolonged exposure to such a poison is certainly one of many anthropogenic activities that has led to 95% of the reefs in the Indo-Pacific to be labeled at risk for degradation and loss (Burke et al., 2011). To combat this threat, governments, environmental groups, and NGOs have often cited the need for the development of a simple, reliable test to detect whether or not a fish has been captured using CN (Bruckner & Roberts, 2008; Davis, Murray & Katsiadaki, 2017). Such a test would allow for the screening of captured fish and potentially the enforcement of existing laws banning this practice.

While development of such a test has long been the goal of anti-CN fishing proponents, no test meeting the aforementioned criteria has proven viable for routine use (Breen et al., 2018). A more systematic approach to solving this long-standing problem would be to first investigate the toxicokinetics of CN absorption and its subsequent detoxification and elimination in a variety of marine fish. Indeed, the same groups appealing for a CN test have also routinely cited the need for CN toxicokinetic data on marine fish (Bruckner & Roberts, 2008; Davis, Murray & Katsiadaki, 2017). However, despite this, no published studies have been conducted “concerning the physiology and pharmaco-kinetics of CN^−^ and SCN^−^ with marine fish” (Rubec, Frant & Manipula, 2008).

Cyanide is toxic to all living things. It inhibits the enzyme cytochrome c oxidase, leading to cellular hypoxia and even death. CN is both volatile and rapidly metabolized making it difficult to detect directly. The major metabolic pathway is the conversion to thiocyanate (the anion SCN^−^, SCN) by the enzyme rhodanese provided there are sufficient sulfur donors available. In humans, nearly 80% of CN is metabolized via this pathway (Logue et al., 2010). As marine fish possess the enzyme rhodanese, it is assumed that, as with other species that possess this enzyme, it catalyzes CN metabolism (Day et al., 2018).

When a marine fish is exposed to aqueous CN, it enters the bloodstream either orally (Hall & Bellwood, 1995) and/or through its gills by diffusion (Bruckner & Roberts, 2008). Beyond this, very little is known about CN metabolism in marine fish despite its long history of use for fish capture. However, it has been widely accepted that elevated levels of SCN in the plasma of a marine fish would indicate that the fish was exposed to CN as both CN and SCN have been used as analytical markers for such exposure (Mak, Yanase & Renneberg, 2005; Bhandari et al., 2014). The presence of SCN upon CN exposure has become the focus of more recent studies (Losada & Bersuder, 2017) as SCN has a longer half-life, is more stable and less toxic than CN.

In most mammals the conversion of CN to SCN is rapid, on the order of minutes to hours, while the elimination rate for SCN is somewhat longer, ranging from 4.95 h to 8 days (Logue et al., 2010). A study of juvenile freshwater Rainbow trout (*Oncorhynchus mykiss*) exposed to SCN (39.8 mg/L = 39.8 ppm) directly for 20 days, determined the half-life for elimination of SCN to be 2.02 ± 0.06 days. SCN could be detected up to 8 days, but by day 16, the SCN was below the detection limit (0.5 mg/L) (Brown et al., 1995). Despite the differences in physiology, this elimination rate for SCN is within the range of that reported for SCN elimination in mammalian species upon exposure to CN (Logue et al., 2010). As previously mentioned, it is reasonable to expect marine fish to adhere to mammalian toxicokinetics pathways because the enzyme used to convert CN to SCN in mammals is also present in marine fish (Chew & Ip, 1992). Considering the accepted vertebrate detoxification pathway and the half-life of CN following an acute exposure, any attempt to measure CN concentration directly in marine fish must be carried out soon (hours) after the exposure. While the time interval for measuring CN indirectly via SCN would be longer (days to weeks), exactly how long is not known.

In fact, there has been some debate as to how long it takes for CN and SCN to be eliminated in marine fish (see commentary reported in Bruckner & Roberts (2008)). At the CN detection testing workshop (Bruckner & Roberts, 2008), the opinions of the participants were split into two groups. One group believed CN is quickly metabolized and the SCN produced is excreted in a matter of hours following the mammalian model. The other group believed that CN and its metabolites are retained for longer periods of time because of the infrequent urination of marine fish and their vastly different osmoregulatory system when compared to freshwater fish. While there have been a number of studies on chronic and acute exposures (i.e., LC_50_ 96-h) to CN in both freshwater and marine fish (Davis, Murray & Katsiadaki, 2017), little work has been done simulating CN fishing, that is, a single, short, pulsed exposure (Mak, Yanase & Renneberg, 2005). Additionally, there have been no reports of the half-life of CN or SCN in fish exposed to CN, and hence speculation has continued with little progress made toward developing a viable test.

Freshwater and marine fish are different in many respects, particularly with respect to their uptake and elimination of ions. Freshwater fish have a higher internal osmotic pressure relative to their environment and therefore actively concentrate ions in their tissues and limit the transport (active or passive) of ions out of their body. Marine fish, on the other hand, have lower osmotic pressure than the surrounding water and consequently are at risk of dehydration. Therefore, marine fish must intake large amounts of seawater and concomitantly remove excess ions from their bloodstream (Evans, Piermarini & Choe, 2005; Marshall & Grosell, 2006). To accomplish this osmoregulatory task marine fish have mitochondrial rich ion pumping cells in their gills, termed chloride cells. These cells allow marine fish to drink large volumes of seawater and eliminate monovalent ions from their gills (Evans, Piermarini & Choe, 2005). The kidneys then filter the blood and are the active site for removal of divalent ions producing highly concentrated urine. Consequently marine fish have a high ion turnover facilitated by active transport of monovalent anions across the gills and passive diffusion of sodium and potassium (Marshall & Grosell, 2006). While the kidneys act as an important blood filter, fish do not remove large amounts of monovalent ions via renal function (Pane et al., 2006).

There is a critical need for SCN toxicokinetic data following CN exposure in marine fish. Such data would provide a timeframe over which any future CN test developed would be viable and would reduce speculation as to how the toxicokinetics of CN might be different between marine fish, freshwater fish, and mammals. Here, we report the use of HPLC-UV to detect and quantify SCN in the plasma of the of the Common clownfish (*Amphiprion ocellaris*). This method can be used for detecting SCN in plasma of both fish exposed to CN and fish directly exposed to SCN. By measuring the half-life of this ion in the plasma, the elimination of SCN from both direct exposure and enzymatic conversion can be compared. Two different experiments were conducted. In the first experiment, *A. ocellaris* were exposed to a solution of 50 ppm CN for either 20, 45, or 60 s, allowed to recover and held in tanks for depuration for up to 41 days. In the second experiment, *A. ocellaris* were exposed to 100 ppm SCN for 11 days and then held in tanks to depurate for up to 48 days. In both cases, plasma samples were collected post-exposure and analyzed for their SCN concentration to follow the rate of SCN elimination, with the goal of determining the half-life of SCN in a marine fish.

## Methods

### Test species and cyanide sources

All experiments were approved by the Roger Williams University Institute of Animal Use and Care Committee (Approval #R180503). *A. ocellaris* were cultured at Roger Williams University and used for both experiments thereby ensuring no previous CN or SCN exposure. Fish of similar mass were selected from a cohort that was approximately 1-year-old (Files S1 and S2). In one experiment, fish were exposed to a solution of 50 ppm CN (NaCN, Sigma 380970) in groups of 10–12 (Files S1 and S2). Three exposure times were undertaken: 20, 45, or 60 s. However, because of high mortality for the first 60 s exposure, only one group was dosed (see Results below). In total, 12 fish were exposed for 60 s, 54 fish for 45 s, and 48 fish for 20 s. Following exposure, the fish were rinsed successively in two seawater baths. After rinsing, fish (*n* = 10–12) were housed in round, 20 L polycarbonate tanks containing filtered seawater (salinity = 32) with light aeration. Temperature was maintained by a water bath held at 26 °C. All fish were fed pelletized food (Ken’s Tropical Green Granule 1 mm) once per day. Water quality was maintained through daily water changes (100%). For the second experiment, fish were exposed to 100 ppm SCN (NaSCN, Sigma 467871) for 11 days. Fish were housed in three separate round, 20 L polycarbonate tanks holding 11 fish each. The SCN concentration was maintained by adding 21.5 mL of a 1.20M solution of SCN to 15 L of seawater during daily water changes. Upon depuration, the fish were rinsed in four consecutive seawater baths to ensure any residual SCN was washed off, and then transferred by treatment group to three separate depuration tanks. Tanks and fish were maintained as above.

### Plasma extraction and hematocrit

Fish were removed from the holding tanks for plasma collection at approximately 1, 2, 4, 8, etc. hours with exact times found in S1 and S2. Fish were anesthetized with tricaine methanesulfonate (Western Chemical Inc., Ferndale, WA, USA) at a concentration of 200 ppm, buffered 2:1 with sodium bicarbonate in seawater. The fish were weighed on an analytical balance and the blood samples were collected by severance of the caudal peduncle with a #21 surgical blade. The blood was collected in 40 mm heparinized microhematocrit tubes (Jorvet, Loveland, CO, USA) and then centrifuged (ZipCombo Centrifuge, LW Scientific, Lawrenceville, GA, USA) at 3,000 rpm for 2 min followed by 6,000 rpm for 5 min to separate the red blood cells from the plasma. The tubes were snapped at the plasma and red blood cell interface and the plasma was aspirated from the capillary tubes into pre-weighed 1.7 mL centrifuge tubes and then re-weighed to determine the mass of plasma collected. The plasma samples were frozen at −80 °C until they were analyzed for SCN by HPLC. After the centrifugation and prior to aspiration, the microhematocrit tubes were photographed for hematocrit (HCT) analysis. Hematocrit is the volume percentage of red blood cells and was measured from the digital images of the levels of the red blood cells to the total volume collected as observed in the HCT tubes using ImageJ. We used a 95% confidence interval to determine if there was a treatment effect, CN exposure, on HCT profile (Riffenburgh, 2012).

### Protein precipitation

Just prior to analysis by HPLC, cold HPLC grade acetonitrile was added to the plasma in the ratio of 1:5. The solution was vortexed for 20 s and then centrifuged at 12,000 rpm for 10 min. The supernatant was pipetted to a new 1.7 mL centrifuge tube and the acetonitrile was evaporated with nitrogen gas in an AutoVap (Zymark, Midland, ON, CA) at 70 °C. The sample was reconstituted in enough HPLC grade water for a 1:5 or 1:10 dilution based on the volume of the plasma collected and days post-exposure (DPE). Samples were then vortexed for 20 s, filtered and injected into the HPLC.

### Thiocyanate analysis

Thiocyanate standards, both in water and in salmon plasma (purchased from Mybiosource Inc., San Diego, CA, USA) and plasma samples were analyzed by HPLC-UV (Rong, Lim & Takeuchi, 2005) using a Waters 515 pump, a Jasco UV-1575 single wavelength detector operating at 220 nm and a Peak Simple data acquisition system (SRI Instruments, Torrance, CA, USA). The column (Devosil 5 µm C_30_-UG, 150 × 4.6 mm id) was treated with 5% polyethylene glycol (PEG). The column was rinsed with the PEG solution for 30–60 min with a flow rate of 0.3 mL/min until the pressure exceeded 2,000 psi and then rinsed with the mobile phase for 1 h or until the pressure stabilized prior to each analysis. The mobile phase consisted of 350 mM sodium sulfate. Injections were done manually using a Rheodyne 7125 injector with a five µL sample loop. Analytical standard solutions of SCN (10.0 ppb–25.0 ppm) prepared in both HPLC grade water and also in commercially available salmon plasma were used to generate calibration curves. Five-point calibration curves were prepared prior to all plasma analysis, such that the concentration of standards bracketed the expected plasma concentration. Over the course of the 3 months that samples were analyzed, the retention time of SCN in the standards was found to range from 5.1 to 5.3 min.

The SCN half-lives and the accompanying regression statistics were determined using Origin 2018 (OriginLab, Northampton, MA, USA). The data were fit using the exponential fitting tool and fit to a single exponential decay function, $\left( {y = A1*{{\rm{e}}^{ - x/{t_1}}} + {y_0}} \right)$, where *x* is time, *y* is concentration, *y*_0_ is the value of the function at infinite time, that is, the asymptotic limit, *A*1 is the concentration maximum, and *t*_1_ is the reciprocal of the first-order elimination rate constant *k*. None of the variables were constrained and no weighting function was used. Fits were to the full data set, the plasma concentration for each fish measured was treated as an individual sample point, and were not averaged at each sampling interval. The reported half-life is related to the rate constant by ${t_{1/2}} = {{{\rm{ln}2}} \over k}$.

## Results

### Cyanide exposure

Upon exposure to a 50 ppm solution of CN, the fish behaved erratically, initially darting about after 30 s in the CN bath, before the onset of paralysis (∼45 s). This behavior was not observed in the 20 s exposure. Regardless of exposure time, all fish lost balance and were paralyzed in the recovery tank. The paralysis time varied proportionally to the exposure time. The longer exposure time (60 s) showed a much higher mortality rate (41.6%) and longer recovery time (27 min) than 45 s which had a 9.8% mortality and an 18 min recovery time. The 20 s exposure had no mortality and an 8 min recovery time. One group of fish (g5) in the 45 s treatment were exposed for longer than 45 s. This group was excluded from any further analysis. The average mass of all fish exposed was 5.29 g (s.d. 1.47), suggesting that 60 s exposure to 50 ppm CN approaches the LC_50_ for this species and size. All exposed fish refused food for the first 3 days, but then ate regularly.

**Figure 1:**
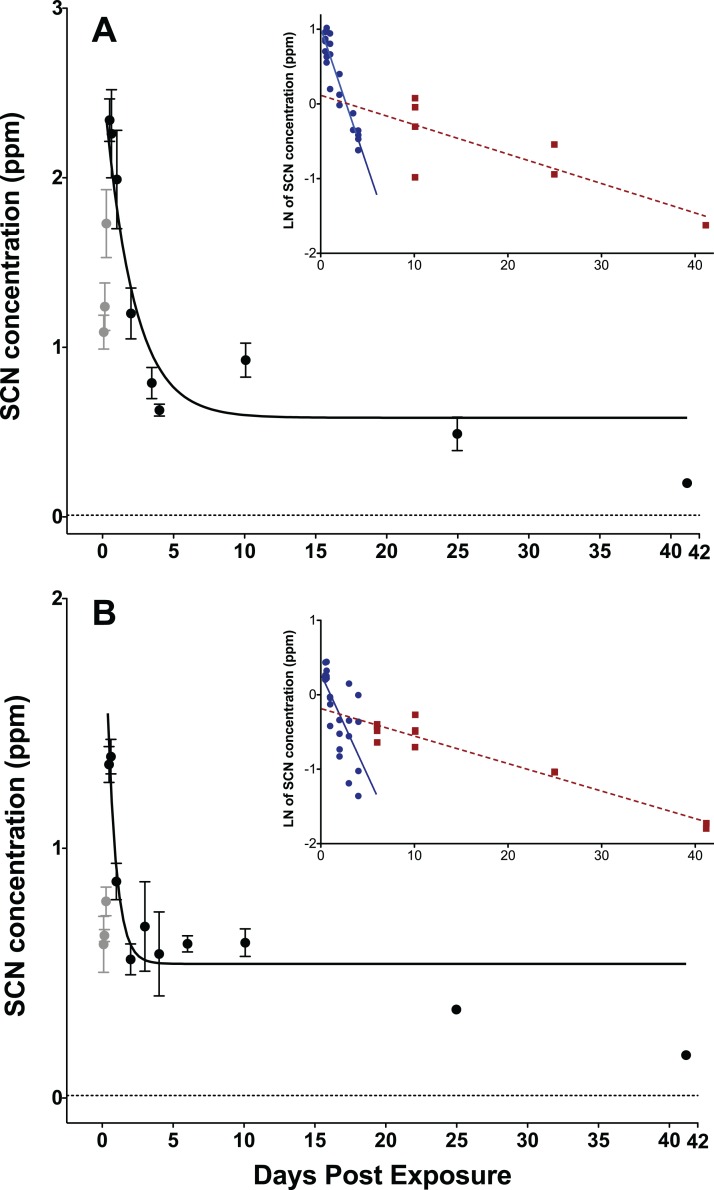
Depuration curves for plasma SCN concentration in *Amphiprion ocellaris* after exposure to 50 ppm cyanide (CN) for (A) 45 s and (B) 20 s. In both (A) and (B) the black solid line represents the fit of the data to a single phase exponential decay function. Gray data points represent early time points where SCN plasma levels are increasing and are therefore not included in the fit. Each data point indicates the mean ± S.E. The dashed gray line represents the detection limit. Where no error bar is observed the error is smaller than the data point. Insets are natural log of plasma SCN concentration as a function of time. These data were divided into two groups and linear fits were performed on both the fast (round/solid) and slow (square/dashed) component of SCN elimination. See Table S1 and File 1 for the data of one treatment batch (group 5, 45 s exposure) excluded from plots.

After exposure to CN, the SCN concentration in the plasma was observed to increase quickly and then began to decrease rapidly during the elimination phase. A maximum in the SCN was observed 12 h post-exposure corresponding to a concentration of 2.3 ± 0.2 ppm for the 45 s exposure and 1.9 ± 0.6 ppm for 20 s exposure (Fig. 1; Table S1). Typical chromatograms are shown in Figs. S1 and S2 of the Supplemental Material. The SCN levels in the plasma did not return to the level observed in the controls but remained elevated, even after 41 days. Control fish showed either no peak attributable to SCN (i.e., 0 ppb), or features so small (<10 ppb in diluted plasma, or correspondingly <50 ppb when corrected for 5× dilution) they could not be quantified by our method.

The rate of SCN elimination was fit to a single-phase exponential decay function with time constant parameters using Origin (Table 1). Regression statistics demonstrated both exposures to be statistically significant when fit to a single-phase exponential decay function (45 s: *r*^2^ = 0.832 and *P* < 0.0001, 20 s: *r*^2^ = 0.686, *P* < 0.0001). The data were fit without constraining *y*_0_, and thus the concentration of SCN was not forced to go to zero at infinite time and resulted in *y*_0_ values of 0.61 ± 0.10 ppm for 45 s and 0.54 ± 0.06 ppm for 20 s exposures. Over the first five DPE a fast, initial decrease of the SCN concentration in the plasma was observed, however, SCN did not return to control levels, but rather remained elevated above controls even up to 41 DPE. This suggests there is likely a second, much slower elimination mechanism present, which has a half-life on the order of days to weeks. Attempts to determine both a fast and slow half-life by fitting the data in Origin to a two-phase exponential decay function did not converge. Instead, in order to make an estimate of the slower half-life, a graph of the natural log of concentration vs. time was constructed using Origin, and the data were divided by inspection into a fast or early time component (blue) and a slow or later time (red) component (Fig. 1 inset). Each component group was fit to a linear function and the half-life was calculated from the resultant slope of the linear fit (Fig. 1 inset). In this manner, the half-lives for the slow component were determined to be 22 ± 5 days for the 45 s exposure and 19 ± 2 days for the 20 s exposure.

**Table 1:** Analysis of the plasma thiocyanate kinetics in *Amphiprion ocellaris* exposed to 50 ppm cyanide.

Parameter	45-s exposure	20-s exposure
*t*_1/2_ (days)	1.01 ± 0.26	0.44 ± 0.15
*Y*_0_ (ppm)	0.609 ± 0.097	0.536 ± 0.055
*A*1 (ppm)	2.54 ± 0.29	1.88 ± 0.60
*t*_1_ (days)	1.45 ± 0.37	0.63 ± 0.22
*k* (days^−1^)	0.69 ± 0.17	1.59 ± 0.56
*r*^2^	0.832	0.686
*P*	*P* < 0.0001	*P* < 0.0001

**Note:**

Results including standard errors of the fit to the function $y = A1*{{\rm{e}}^{ - x/{t_1}}} + {y_0}$ of the plasma SCN concentrations in *A. ocellaris* following depuration after exposure to 50 ppm CN. *k* and *t*_1/2_ are calculated from the fit parameter *t*_1_.

### Thiocyanate exposure

**Figure 2:**
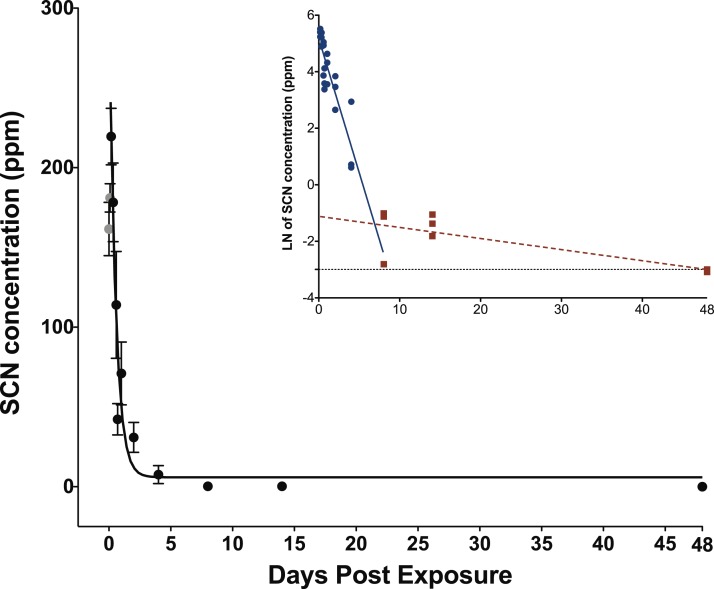
Depuration curve for plasma SCN concentration in *Amphiprion ocellaris* after exposure to 100 ppm SCN for 11 days. The black solid line represents the fit of the data to a single phase exponential decay function. Gray data points represent early time points where SCN plasma levels are increasing and are therefore not included in the fit. Each data point indicates the mean ± S.E. The dashed gray line represents the detection limit. Where no error bar is observed the error is smaller than the data point. Inset is natural log of plasma SCN concentration as a function of time. These data were divided into two groups and linear fits were performed on both the fast (round/solid) and slow (square/dashed) component of SCN elimination.

When exposed to SCN, the fish behaved normally, ate regularly and did not show any signs of stress. Upon depuration, the SCN level in the plasma of fish exposed to 100 ppm of SCN for 11 days reached a maximum of 204 ppm 4 h after depuration began and then decreased rapidly (Fig. 2; Table S2). SCN elimination was well approximated by a single exponential decay (*r*^2^ = 0.84, *P* < 0.0001) (Table 2). However, as in the case for CN exposure, while there is an initial rapid SCN elimination, there is also a slower component as the SCN concentration in the plasma did not return to zero or near control levels after 14 days. At 48 days, the last time period sampled, control levels were reached. As was the case for CN exposure, a graph of the natural log of SCN concentration vs. time was constructed, and the half-life from the linear fit of the data for the slower component was found to be 18 ± 5 days.

**Table 2:** Analysis of the plasma thiocyanate (SCN) kinetics in *Amphiprion* *ocellaris* exposed to 100 ppm SCN for 11 days.

Parameter	Value
(*t*_1/2_)_1_ (hours)	0.35 ± 0.07
*Y*_0_ (ppm)	8.52 ± 9.7
*A*1 (ppm)	305 ± 37
*t*_1_	0.507 ± 0.097
*k*_1_ (hours^−1^)	1.97 ± 0.38
*r*^2^	0.84
*P*	*P* < 0.0001

**Note:**

Results including standard errors of the fit to the function $y = A1*{{\rm{e}}^{ - x/{t_1}}} + {y_0}$ of the plasma SCN concentrations in *A. ocellaris* following depuration after exposure to 100 ppm SCN for 11 days. *k* and *t*_1/2_ are calculated from the fit parameter *t*_1_.

### Hematocrit data

The observed HCT data (Table 3) is consistent with CN exposure. HCT was suppressed (below 95% CI of controls) for the first 36 h for the 45 s exposure and the first 24 h for the 20 s exposure. As elimination of SCN from the plasma proceeded, HCT levels began to recover to near control levels of 50% ± 6%. SCN exposure also reduced HCT levels but their response was more variable with HCT levels increasing above the control levels by eight DPE.

**Table 3:** Hematocrit profile of *Amphiprion ocellaris* following exposure to thiocyanate (SCN) or cyanide (CN).

Time post-exposure (days)	Hematocrit post SCN exposure	Hematocrit post 20 s CN exposure	Hematocrit post 45 s CN exposure
0.090	42% (11%)^†^	38% (3%)^†^	36% (4%)^†^
0.18	46% (4%)	36% (1%)^†^	36% (1%)^†^
0.27		38% (2%)^†^	37% (2%)^†^
0.34	36% (8%)^†^		
0.49		37% (4%)^†^	38% (1%)^†^
0.59	32% (1%)^†^		
0.70	32% (3%)^†^		
1.0	43% (1%)	37% (2%)^†^	36% (2%)^†^
2.0	50% (<1%)	43% (5%)	36% (3%)^a^^†^
3.0		44% (3%)	40% (1%)^†^
3.5			48% (8%)^b^
4.0	40% (4%)^†^	43% (7%)	42% (4%)^†^
6.0		49% (4%)	51% (2%)
8.0	62% (5%)^†^		
10		44% (2%)	47% (5%)
Control	50% (6%)		

**Notes:**

Fish were exposed to 100 ppm SCN for 11 days or 50 ppm CN for 20 or 45 s. Values given as means (SD), sample size of 3 in SCN exposures and 4 in CN exposures and six controls. Exclusions noted.

a Sample size of 3 at 2.0 days post 45 s CN exposure.

b Sample size of 2 at 3.5 days post 45 s CN exposure.

† Sample fall outside of 95% CI of control.

## Discussion

### CN exposure

Both the 45 s and the 20 s exposure times exhibited a maximum plasma SCN concentration at 12–15 h post-exposure. The 45 s exposure time produced a higher concentration maximum and a longer half-life than the 20 s exposure when comparing the results of the single-phase exponential fits. The maximum observed in the SCN plasma concentration at the 45 s exposure is very close to that observed for the limited data we have at 60 s exposure. As there was a much higher mortality rate observed for the 60 s dose than the 45 s dose of 50 ppm CN, the amount of CN absorbed for the 60 s exposure is likely near the LC_50_. This suggests that plasma SCN concentration would likely not increase even with a greater CN dose, but rather only the mortality rate would increase. While the maxima observed for the 45 and 60 s exposure SCN concentrations were very close, they were nearly twice that observed for the 20 s exposure, indicating that the SCN concentration has leveled off, despite the increased dose. A similar dose-dependent response has been observed in rats (Bhandari et al., 2014). The rise and then leveling off of the SCN plasma concentration with increasing dose could indicate that there is a shortage of sulfur donors needed for conversion to SCN and that the pathway for enzymatic conversion is saturated at the higher doses (Jacobson-Kram & Keller, 2006; Day et al., 2018).

The variation in the maximum SCN concentration observed in the plasma is easily understood as a dosing effect, but the difference in the half-lives is puzzling as true first-order kinetics should be independent of initial concentration. However, this is also likely a dosing effect. SCN is itself a metabolite and is being generated as it is being eliminated. For a much heavier and near-lethal dose, it takes longer for the fish to recover and convert all of the CN present to SCN. This would particularly be the case if the conversion rate was limited by a lack of sulfur donors (Day et al., 2018).

The half-lives determined from the exponential fits in Origin for SCN elimination upon CN exposure in *A. ocellaris* are relatively fast and are in reasonable agreement with those reported for rats, pigs, and goats, but are at the faster end of the range of times reported for humans (Logue et al., 2010). However, while these half-lives are in the range of what is reported for mammals, the SCN levels in *A. ocellaris* do not return to control levels after long periods of times (41 days) as is the case for mammals. The half-lives reported here represent the time it takes to reach the asymptotic limit, *y*_0,_ (see equation used for fit in Methods). *y*_0_ is the concentration of SCN remaining at infinite time. In our fit, it was not constrained to be zero at infinite time and from the results of the fit it is seen that a substantial amount of SCN remains in the plasma even after many half-lives. Following first-order kinetics, after five half-lives there should only be about 5% of the initial SCN present remaining. The longest half-life we report is 1.01 days for the 45 s exposure to CN. If all SCN was eliminated by one mechanism with this half-life following first-order kinetics, then by 5.0 DPE all the SCN in the plasma should be eliminated or have dropped below our detection limit. However, the fish examined show SCN plasma levels remaining in the hundreds of ppb even up to 41 DPE. This is indicative of a second elimination mechanism with a slower (18–22 days) half-life that is limiting the rapid and complete elimination of SCN. This is not seen in mammalian pathways, however, this could be due to a much higher endogenous level of SCN in mammals masking the presence of a slower mechanism (Bhandari et al., 2014). The fast mechanism, whose half-life we measure with our exponential fit, is likely due to an active exchange through the gills where monovalent anions like SCN are readily excreted (Evans, Piermarini & Choe, 2005). The slower mechanism, whose half-life we measure from our semi-log fits, could reflect the time it takes for CN bound to either metals, hemoglobin or proteins, that is, the CN in the CN pool, to be released from its bound state and subsequently converted to SCN. As most CN in the blood is either charge neutral (HCN) or bound to hemoglobin, it would not be excreted as efficiently through the gills as SCN, a monovalent anion. Initially, when there are high levels of free CN and ample sulfur donors, CN is rapidly converted to SCN and excreted. Our data suggest that once the sulfur pool and/or the free CN is depleted rapid conversion to SCN stops. Conversion slowly resumes as the sulfur pool (Day et al., 2018) is replenished or as HCN is released back into the bloodstream from its bound state. It will then be excreted, but this is a much slower process resulting in SCN concentrations remaining above control levels for much longer time than the single exponential model would predict. The presence of SCN above control levels in the plasma of *A. ocellaris*, days to weeks after CN exposure is a promising indication that this could serve as a suitable marker for CN exposure.

The diffuse nature of the supply chain of wild caught marine aquarium fish make determining the time between capture and import challenging. From collection to final destination marine ornamental fish pass through a variable, fragmented, and complex supply chain that could take anywhere from 3 days to 2 weeks (Amos & Claussen, 2009; Cohen, Valenti & Calado, 2013). To use this method as a CN detection test in the importing country, SCN would need to remain above the detection limit and/or control values for weeks. If SCN does not remain elevated, there would little chance of using this method as a marker for CN exposure unless testing was done in the country of origin very soon after capture. If the dose *A. ocellaris* received in our study is typical of that used in CN capture, and if the presence of the slower, second mechanism is present in all marine fish, then it might be possible to use this method for a CN exposure test.

### SCN exposure

The concentration of SCN in the plasma of fish exposed to 100 ppm SCN had a maximum of 204 ppm 4 h after depuration started. This concentration is more than twice that of the treatment dose, but this is reasonable for chronic exposure to SCN (Brown et al., 1995). The 4-h delay for plasma SCN to reach a maximum may be the result of the physiological changes in osmoregulation that occur as SCN is removed from the external environment. The SCN that accumulated throughout the fish during uptake is released to the plasma as depuration begins and the physiological processes within the gills adjust to accommodate the absence of SCN in the bath (Brown et al., 1995). Note, we did not sample at *t* = 0, but rather three fish were bled at 2, 7, and 12 min, respectively, reflecting the time it took for the seawater rinses and to bleed each fish (S1 and S2).

The half-life of 0.35 ± 0.07 days and the presence of an appreciable SCN concentration over a long time is in good agreement with what we observed for our 20 s CN exposure. The similar half-lives suggest that chronic exposure to SCN could be used as a proxy for exposure to CN to determine the rate of elimination of SCN in marine fish. Accordingly, a half-life determined from SCN exposure would not be limited by the lack of sulfur donors, but would more likely reflect the true rate that the active pumping mechanism through the gills can excrete the SCN. Indeed, the SCN exposure half-life is slightly faster than what was observed for the 20 s CN exposure as there is no chemical transformation that must occur before elimination. While SCN plasma levels do return to control levels at our last time point measured, 48 days, there is still appreciable SCN in the plasma at 14 days (Table S2). Here, the presence of SCN above what would be expected for a single exponential decay is likely due to slower, diffusion-limited processes, representing the time it takes for SCN to diffuse back into the plasma from its initial deposition in tissues throughout the fish (Toutain & Bousquet-Mélou, 2004; Jacobson-Kram & Keller, 2006). For this chronic exposure, SCN should be deposited throughout all tissues in the fish, not just those tissues with a high blood flow (e.g., liver, spleen, kidney, gills).

The 0.35-day half-life from the exponential fit in this study is faster than the 2.09 days reported for the freshwater fish Juvenile trout (*O. mykiss*) suggesting that the active mechanism for anion removal is much faster in marine fish than in freshwater fish. Given the differences in osmoregulation of freshwater and marine fish, and the ability of marine fish to excrete monovalent ions quickly through the gills, a faster half-life is reasonable (Marshall & Grosell, 2006). Brown et al. (1995) did not observe a long residence time in freshwater rainbow trout that we observed in *A. ocellaris*, but the higher detection limit reported for that study might have prohibited them from observing this behavior.

## Conclusion

Thiocyanate in the plasma of the marine fish *A. ocellaris* is easily quantified using HPLC-UV and C30 column pre-treated with PEG. SCN can be observed in the plasma of *A. ocellaris* following exposure to CN or SCN. Plasma SCN levels observed appear to be CN dose-dependent, reflecting the longer time it takes for conversion to SCN the heavier the dose of CN. The SCN exposure half-life is in reasonable agreement with the half-life measured for the lower dose of CN exposure (20 s) suggesting that chronic exposure to SCN can be used as a proxy for measuring the rate of SCN elimination. Any half-life reported for the depuration of SCN following exposure to SCN would likely represent the fastest rate of elimination possible.

Historically, there has been disagreement as to how fast marine fish would excrete CN or SCN. Would these rates be fast, in accordance with mammalian models, or would they be slower in marine fish (Rubec, Frant & Manipula, 2008)? Our results support an initial fast elimination mechanism followed by a much slower mechanism as evidenced by long residence times of low levels of SCN. We speculate that variation observed in the fast half-life (0.3–1.0 days) is due to the depletion of the sulfur donors at high doses, and that this rapid elimination is due to an active ion pumping mechanism of monovalent anions through the gills. The slower half-lives are in reasonable agreement with each other within their respective uncertainties (18–22 days) and likely results from a diffusion limited mechanism. In all cases, our reported half-lives are faster than what is observed for the freshwater Rainbow trout (*O. mykiss*). This is easily understood in terms of the vast differences in the osmoregulatory burdens of marine vs. freshwater fish.

The half-lives reported here are for a single species of marine fish, which have been cultivated in a laboratory. The species is believed to have a high tolerance for CN and may not represent the toxicokinetics of all or even most marine fish. Nevertheless, this represents a first attempt to understand the toxicokinetics of CN exposure in marine fish through direct measurement of SCN. However, many more species need to be studied before wide-ranging conclusions can be drawn. Additionally, endogenous levels of SCN in wild caught Indo-Pacific marine fish need to be surveyed to measure baseline levels and minimize false positives. The presence of measurable levels of SCN for days to weeks after CN exposure in the plasma of this species above that of the control suggests plasma SCN could serve as a viable marker for marine fish exposed to CN in the capture process.

## Supplemental Information

10.7717/peerj.6644/supp-1 Supplemental Information 1 Supplemental Information

10.7717/peerj.6644/supp-2 Supplemental Information 2 Cyanide exposure data.

10.7717/peerj.6644/supp-3 Supplemental Information 3 Thiocyante exposure data.

## Additional Information and Declarations

### Data Availability

The following information was supplied regarding data availability: The raw data on fish exposure, peak area, and other details are available in the Supplemental File. File 1 contains the SCN exposure and the cyanide exposure.
