# Supplementary material for: On the half-life of thiocyanate in the plasma of the marine fish Amphiprion ocellaris: implications for cyanide detection
Source: PeerJ. 2019 Apr 2;7:e6644. doi: 10.7717/peerj.6644 (PMC6450372; doi:10.7717/peerj.6644)
Supplement: Supplemental Information 1 [file peerj-07-6644-s001.docx]

**SUPPLEMENTAL INFORMATION**

**On the half-life of Thiocyanate in the plasma of the marine fish Amphiprion ocellaris: implications for cyanide detection**

Nancy E. Breen^1^, J. Alexander Bonanno^2^, Sara Hunt^1,3^, Julia Grossman^3^, Jordan Brown^1^, Hannah Nolte^1,3^, and Andrew Rhyne^3^

^1^Department of Chemistry and Physics, Roger Williams University, Bristol, RI, USA

^2^School for the Environment, UMass Boston, Boston, MA, USA

^3^Department of Biology, Marine Biology and Environmental Science^,^ Roger Williams University, Bristol, RI, USA

Corrresponding Author:

Andrew Rhyne^3^

Email Address: [arhyne@rwu.edu](mailto:arhyne@rwu.edu)


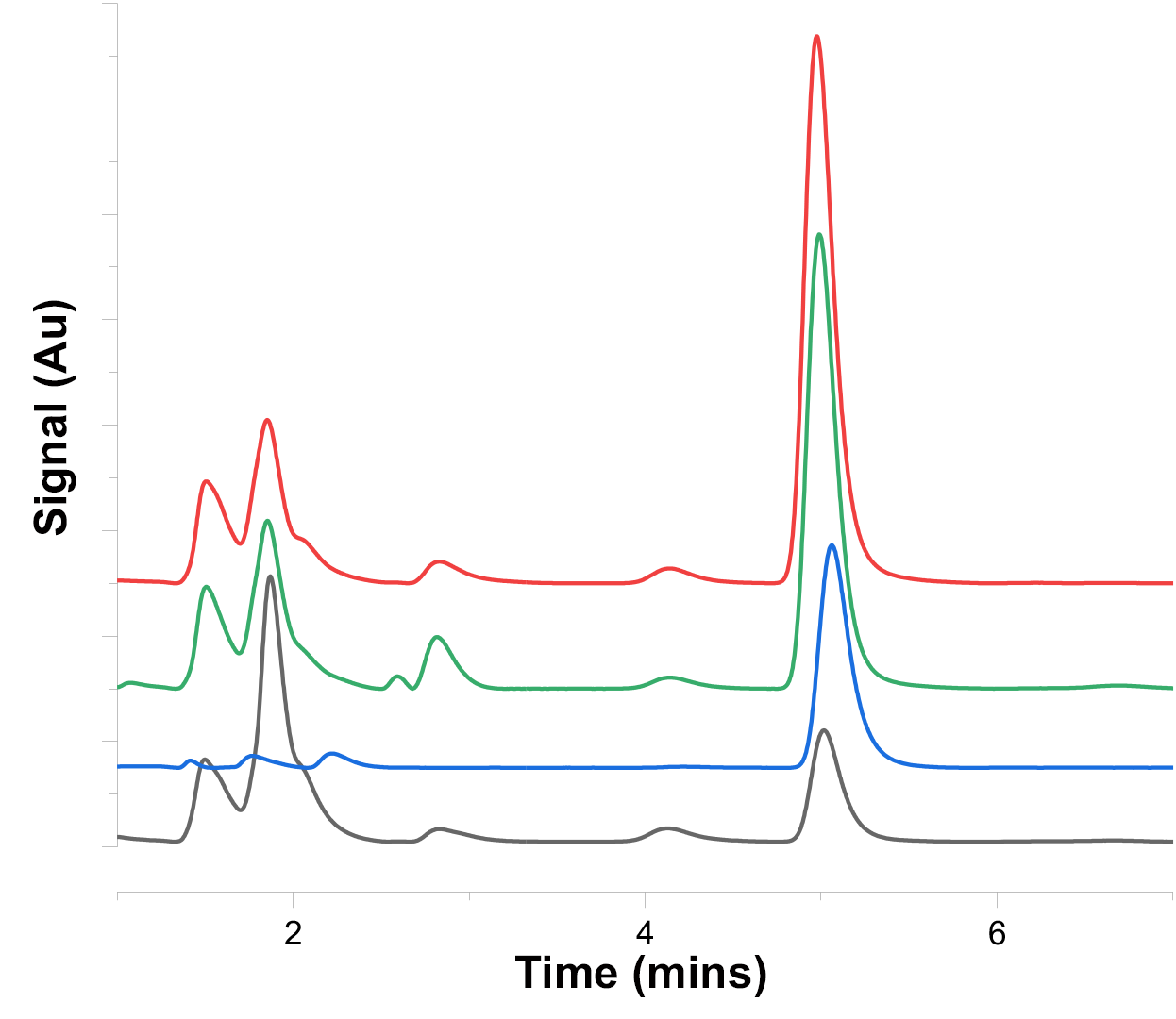


Figure S1. Chromatograms of SCN in the plasma of *Amphiprion oscellaris* after exposure to 100 ppm SCN for 11 days. Depuration times from top to bottom: 2 hours, 8 hours, 10 ppm standard and 49 hours. SCN peak is observed at 5.0 minutes


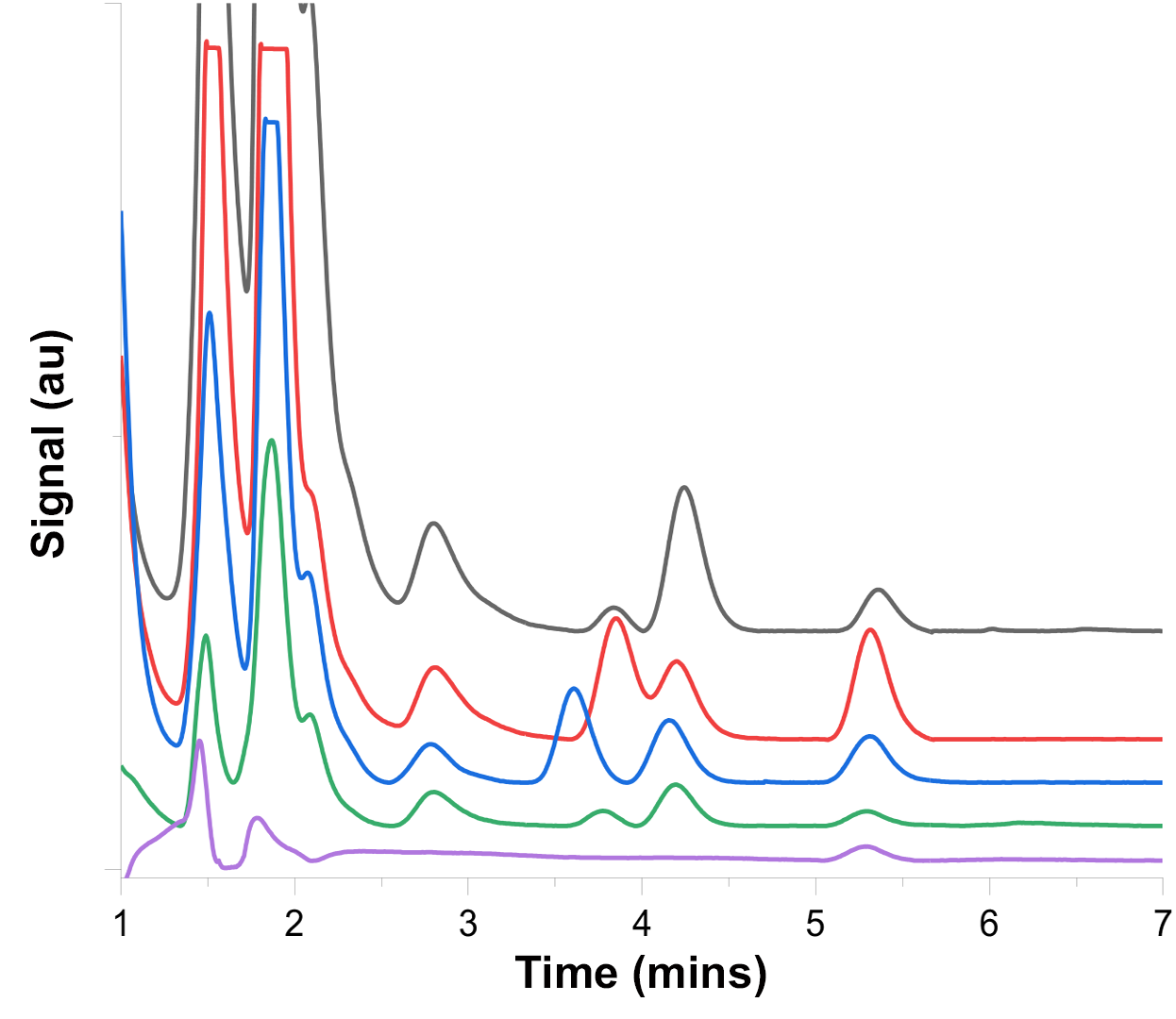


Figure S2. Chromatograms of SCN in the plasma of *Amphiprion oscellaris* after exposure to 50 ppm CN for 45 seconds. Depuration times from top to bottom: 2 hours, 15 hours, 24 hours, 48 hours and 0.1 ppb standard. SCN peak is observed at 5.3 minutes

| Table S1. Mean (SD) Plasma SCN concentrations in *A. ocellaris* exposed to 50 ppm CN for 20, 45 and 60 seconds followed by 41 days depuration. | | | |
| --- | --- | --- | --- |
| Depuration  Time (days) | Plasma SCN concentration (ppm) | | |
|  | 20 second exposure | 45 second exposure | 60 second exposure |
| 0.10 | 0.6 (0.2) | 1.1 (0.2) |  |
| 0.16 | 0.65 (0.05) | 1.2 (0.3) |  |
| 0.27 | 0.8 (0.1) | 1.7 (0.4) | 1.6 (0.4)^a^ |
| 0.49 | 1.3 (0.1) | 2.3 (0.2) | 1.8 (0.3)^a^ |
| 0.62 | 1.4 (0.1) | 2.3 (0.5) |  |
| 1.0 | 0.9 (0.1) | 2.0 (0.6) | 2.0 (0.4)^b^ |
| 2.0 | 0.6 (0.1) | 1.2 (0.3)^b^ |  |
| 3.0 | 0.7 (0.4) | 2.5 (0.4) ^c^ |  |
| 3.5 |  | 0.8 (0.1)^a^ |  |
| 4.0 | 0.6 (0.3) | 0.63 (0.07) |  |
| 6.0 | 0.62 (0.06) | 0.83 (0.08) ^c^ |  |
| 10 | 0.6 (0.1) | 0.8 (0.3) |  |
| 25 | 0.354 (0.002)^a^ | 0.5 (0.1)^ac^ |  |
| 41 | 0.172 (0.009)^a^ | 0.3 (0.1)^ac^ |  |
| Control | <0.05^d^ | | |
| Note.n = 4. Exclusions noted.  ^a^ Sample size of 2 at: 25 and 41 days post 20 and 45 second exposure, at 3.5 days post 45-second exposure and at 0.27 and 0.49 days post 60-second exposure.  ^b^ Sample size of 3 at: 2.0 days post 45 second exposure and at 1.0 days post 60-second exposure.  ^c^ Data from fish in one exposure group (g5) were elimated from analysis due accidental prolonged exposure.  ^d^ Controls were below the LOQ of 10 ppb for 1:5 diluted plasma. | | | |

| Table S2. Plasma SCN concentrations in *A. ocellaris* exposed to 100 ppm SCN for 11 days followed by 48 days depuration. | |
| --- | --- |
| Time post exposure (days) | Plasma SCN concentration (ppm) |
| 0.010 | 162 (29) |
| 0.090 | 181 (13)^a^ |
| 0.18 | 220 (31) |
| 0.35 | 178 (43) |
| 0.59 | 114 (58) |
| 0.70 | 42 (17) |
| 1.0 | 71 (34) |
| 2.0 | 31 (16) |
| 4.0 | 8 (10) |
| 8.0 | 0.2 (0.2) |
| 14 | 0.26 (0.09) |
| 48 | <0.05 |
| Control | <0.05^b^ |
| Note. Values are given as means (SD), all with sample size of 3. Exclusion noted.  ^a^ Sample size of 2 at 0.090 days post exposure.  ^b^ Controls were below the LOQ of 10 ppb for 1:5 diluted plasma. | |
